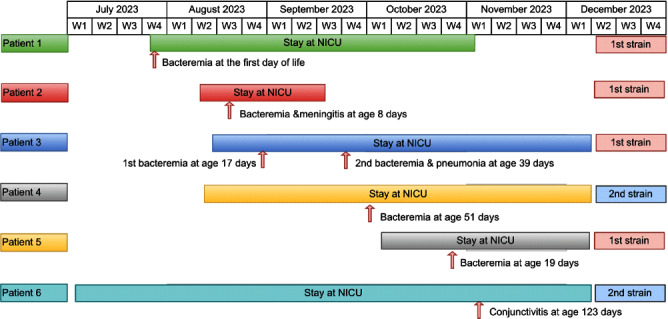# Whole Genome Sequencing for the Identification of a Streptococcus agalactiae Outbreak in Neonatal Intensive Care Unit

**DOI:** 10.1017/ash.2024.283

**Published:** 2024-09-16

**Authors:** Halima Dabaja Younis, Allison McGeer, Vanessa Allen, Angelie Seguban, Andrea Morillo, Irene Martin, Alyssa Golden, Jennie Johnstone

**Affiliations:** Sinai Health System- Toronto; National Microbiology Laboratory, Public Health Agency of Canada; Public Health Agency of Canada - National Microbiology Laboratory

## Abstract

**Background:** While Streptococcus agalactiae (Group B Streptococcus [GBS]) infections in infants usually result from maternal transmission, healthcare-associated cases, particularly in the neonatal intensive care unit (NICU), can occur. Whole genome sequencing (WGS) can aid in investigating GBS outbreaks among infants in hospital settings. The aim of the study is to describe the investigation of GBS infections in NICU using WGS. **Methods:** Infection prevention and control (IPAC) at our hospital monitors the occurrence of late-onset GBS disease (LOD) in our 57-bed NICU, which consists of all private rooms. The occurrence of 2 cases of LOD within 2 weeks triggered an investigation, including WGS of the two isolates and isolates causing invasive GBS during the last 6 months in the unit. GBS isolates underwent WGS using Illumina at Canada’s National Microbiology Laboratory. All affected patients underwent chart-review. Outbreak description and investigation: In August 2023, two NICU neonates (patients 2,3) experienced LOD two weeks apart, one with bacteremic meningitis and the other with two bacteremic episodes three weeks apart. While WGS was pending two additional cases of late-onset GBS bacteremia (patients 4,5) occurred. Isolates from Pts 2,3 and 5 were indistinguishable from each other and from an isolate from an infant admitted to the NICU with early onset bacteremia on July 27, 2023 (day 1 of life) (patient 1). Weekly point prevalence for throat and rectal colonization over 3 weeks identified five infants colonized with unrelated strains. An additional long-stay infant (patient 6) developed GBS conjunctivitis due to a strain indistinguishable from (patient 4) by pulse field gel electrophoresis, WGS for the second cluster is pending. IPAC interventions: Lapses in IPAC practices were observed, with no commonalities among cases other than similar geographic location within the unit. We hypothesized transmission was due to horizontal transmission between babies due to these lapses. Basic IPAC measures, including hand hygiene and environmental cleaning, were reinforced; Additional Precautions were not used due to private rooms’ unit structure. No environmental samples were taken due to lack of an obvious environmental point or common source. Point prevalence monitoring persisted until no new cases related to the outbreak strains were further identified in three consecutive weekly point prevalence. **Conclusions:** Increased awareness of healthcare-associated transmission is crucial in NICU as LOD GBS emerges. WGS plays a key role in identifying transmission. Detecting a multi-strain outbreak can appropriately redirect investigations. Legend: Figure 1: Timeline of stay at NICU and infection timing for patients 1-6

**Figure:**